# The utility of *Apc*-mutant rats in modeling human colon cancer

**DOI:** 10.1242/dmm.016980

**Published:** 2014-10-02

**Authors:** Amy A. Irving, Kazuto Yoshimi, Marcia L. Hart, Taybor Parker, Linda Clipson, Madeline R. Ford, Takashi Kuramoto, William F. Dove, James M. Amos-Landgraf

**Affiliations:** 1 McArdle Laboratory for Cancer Research, Department of Oncology, University of Wisconsin-Madison, Madison, WI 53792, USA.; 2 Institute of Laboratory Animals, Graduate School of Medicine, Kyoto University, Kyoto 606-8501, Japan.; 3 Department of Veterinary Pathobiology, University of Missouri, Columbia, MO 65211, USA.

**Keywords:** APC, Allelic series, Animal models, Colorectal cancer

## Abstract

Prior to the advent of genetic engineering in the mouse, the rat was the model of choice for investigating the etiology of cancer. Now, recent advances in the manipulation of the rat genome, combined with a growing recognition of the physiological differences between mice and rats, have reignited interest in the rat as a model of human cancer. Two recently developed rat models, the polyposis in the rat colon (Pirc) and Kyoto Apc Delta (KAD) strains, each carry mutations in the intestinal-cancer-associated adenomatous polyposis coli (*Apc*) gene. In contrast to mouse models carrying *Apc* mutations, in which cancers develop mainly in the small intestine rather than in the colon and there is no gender bias, these rat models exhibit colonic predisposition and gender-specific susceptibility, as seen in human colon cancer. The rat also provides other experimental resources as a model organism that are not provided by the mouse: the structure of its chromosomes facilitates the analysis of genomic events, the size of its colon permits longitudinal analysis of tumor growth, and the size of biological samples from the animal facilitates multiplexed molecular analyses of the tumor and its host. Thus, the underlying biology and experimental resources of these rat models provide important avenues for investigation. We anticipate that advances in disease modeling in the rat will synergize with resources that are being developed in the mouse to provide a deeper understanding of human colon cancer.

## Introduction

Colon cancer is a leading cause of cancer-related death worldwide. Globally, ~1.4 million new cases of colorectal cancer were diagnosed in 2012, making up nearly 10% of all cancers (Ferlay et al., 2014). In the United States, ~130,000 people were diagnosed with colorectal cancer in 2010, and over 50,000 people died from the disease (Centers for Disease Control and Prevention, 2014). Overall, one in every 20 people will develop colon cancer in their lifetime (American Cancer Society, 2014a).

Many animal models of colorectal cancer are based on mutations in the *Apc* (adenomatous polyposis coli) gene, which is mutated in over 80% of colon cancers in humans (Fearnhead et al., 2001). The normal function of this gene includes the negative regulation of signaling by Wingless-Int (WNT) cytokines (see Box 1 for a glossary of terms). In the absence of APC function, the WNT pathway is activated through β-catenin, leading to the transcription of a battery of tumor-promoting genes, including *Myc* (Walz et al., 2014). Animal models that recapitulate human colon cancer can help to improve its management in several complementary ways: by improving our understanding of the etiology of tumor development from the normal colonic epithelium to adenoma to adenocarcinoma; by identifying cancer-detection methods that are accessible and allow early diagnosis; by facilitating the development of chemopreventive strategies; and by developing novel, targeted treatment strategies. The goal of this Review is to highlight the complementary features and utility of two rat models in investigating human colon cancer: the heterozygous polyposis in the rat colon (Pirc) strain and the homozygous Kyoto Apc Delta (KAD) strain. These models closely mimic *Apc*-dependent neoplasia in humans: tumors form more frequently in the colon than in the small intestine, and more frequently in males than in females. The generation of these rat models is summarized in Box 2.

**Box 1. Glossary of terms**

5-FU: 5-fluorouracil. A chemotherapeutic agent that prevents DNA synthesis.
ACI: August Copenhagen Irish; an inbred rat strain.
Adenocarcinoma: an invasive form of an epithelial cancer (no longer an adenoma).
Adenoma: an early epithelial lesion showing neoplastic potential to convert into an adenocarcinoma.
Allele: one of several different forms of any one locus in the genome.
AOM: azoxymethane. A chemical carcinogen whose action is selective for the colon of experimental animals.
*APC/Apc*: *APC* is the human gene and *Apc* is the mouse or rat gene encoding adenomatous polyposis coli (APC), a very large cytoplasmic protein with a number of regulatory interactions.
*Apc^Min/+^* mouse: a mouse model of spontaneous *Apc*-dependent intestinal cancer created by ENU-induced germline mutagenesis. Also known as the Min mouse.
BN: Brown Norway; an inbred rat strain.
Catenin: three isoforms exist – alpha, beta and gamma – and these proteins interact with the cell-adhesion cadherins but translocate to become cofactors in the transcription apparatus.
Celecoxib: a highly selective inhibitor of the cyclooxygenase-2 (COX-2) enzyme. Evidence supports its use as a chemopreventive agent against colon cancer.
Chemoprevention: the use of chemical treatments to prevent the earliest stages in a disease such as cancer.
Coisogenic: a derivative of an inbred strain of mice or rats that carries only the new mutation defining the strain.
Congenic: differs from coisogenic in that it is a derivative of an inbred strain that carries a segment of a donor strain and therefore carries a number of sequences from the donor strain introgressed onto the genome of the recipient strain.
CRISPR/Cas: clustered regularly interspaced short palindromic repeats. A method for the targeted disruption or replacement of the sequence of a chosen gene(s).
CTC: computer tomographic colonography. A radiographic method for monitoring the appearance of colonic tumors in humans. MicroCTC is the same method scaled down to the size appropriate to experimental animals.
DahlSS: an inbred rat strain that shows hypertension in the presence of elevated salt in the diet.
DSS: dextran sodium sulfate. An ulcerogenic and inflammatory agent that promotes tumor formation.
Early carcinoma: a neoplasm that has invaded through the muscularis mucosa and into but not through the submucosa.
EB1: an interactive domain in the C-terminal region of the APC protein.
ELISA: enzyme-linked immunosorbent assay. A very sensitive assay for the level of antigen given a specific antibody directed against the antigen.
ENU: N-ethyl-N-nitrosourea. A potent alkylating agent that is particularly effective in mutating the germline of experimental animals.
F1 hybrid or derivative: a product of mating between two different inbred strains. These F_1_ animals are identical to one another but if bred further they generate progeny that segregate for the alleles that differ between the two parental inbred strains.
F344: Fischer 344; an inbred rat strain.
FAP: familial adenomatous polyposis. A heritable human disease involving hundreds to thousands of colonic polyps, caused by mutation in the human *APC* gene.
Gel-molding: *in vivo* molding of colonic tumors using dental alginate, to determine volume.
Haploinsufficiency: a situation in which there is an abnormal phenotype when there is only one functional copy of a gene in a diploid organism.
hDLG: the interaction domain in the C-terminal region of the APC protein of the human ortholog to the *Drosophila discs large* gene.
Intramucosal carcinoma: a neoplasm that has invaded the lamina propria or into but not through the muscularis mucosa in the intestine.
KAD: Kyoto Apc Delta rat. A rat model isolated in Kyoto, Japan that was created by germline ENU-induced mutagenesis that generates a nonsense mutation in the *Apc* gene leading to tumors only when also exposed to AOM and DSS. Also known as F344-*Apc^m1Kyo^*.
KURMA: Kyoto University Rat Mutant Archive created by ENU mutagenesis of the mouse germline.
Lamina propria: the membrane closest to the epithelium of an intestinal crypt.
MicroCT: computed tomography scaled down to a size appropriate to experimental animals.
Monoallelic: expression of only one of the two alleles in diploids.
MuT POWER: a method from phage Mu that detects new mutations for any chosen gene.
NMR: nuclear magnetic resonance. An imaging method that does not use ionizing radiation.
Odontoma: a benign tumor of early tooth development.
Pirc (*Apc^Pirc/+^*) rat: a rat model of *Apc*-dependent spontaneous intestinal cancer created by targeted ENU-induced germline mutagenesis. Pirc stands for ‘polyposis in the rat colon’ and these rats develop multiple colonic tumors.
Pleiotropy: an effect on many different processes by mutation in one locus.
Polymorphisms: natural variation at any one locus within a species.
TALEN: transcription activator-like effector nuclease. Used in the targeted disruption of a gene(s).
WNT3A: an isoform of the cytokine encoded by the WNT gene family. The WNT abbreviation is short for *wingless*, a gene that controls development of the *Drosophila* wing, and *Int*, the site of tumorigenic insertion of a tumor virus.
ZFN: zinc-finger nuclease. Used for targeted disruption of a gene(s).

**Box 2. Derivation of the Pirc and KAD strains**

**Fig. 1.**
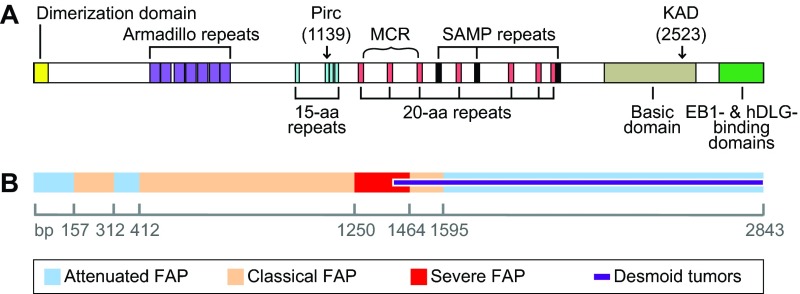
**Human *APC* gene structure and mutations.** (A) Structure of the human *APC* gene. Positions are shown for the dimerization domain (7-aa repeats), the ASEF-binding domain (40-aa Armadillo repeats), the β-catenin-binding domain (15-aa repeats), the β-catenin-degradation domain (20-aa repeats), the axin-binding domain (SAMP repeats), the basic domain (microtubule-binding domain) and C-terminus (EB1- and hDLG-binding domains). Arrows indicate orthologous locations in the human gene of the Pirc and KAD truncating mutations. Curly bracket indicates the location of the mutation cluster region (MCR) for sporadic colon cancer in humans (Kohler et al., 2008). (B) The correlation between mutation site and severity of FAP disease in humans; boundaries are indicated on the ruler (bp) (Nieuwenhuis and Vasen, 2007). aa, amino acid; APC, adenomatous polyposis coli; EB1, end binding 1; hDLG, human homolog of the *Drosophila discs large* gene; SAMP, Ser-Ala-Met-Pro motif.

The *Apc^Pirc/+^* (Pirc) rat was developed by targeted ENU-induced germline mutagenesis (van Boxtel et al., 2010) of the inbred F344/NTac rat strain (Amos-Landgraf et al., 2007). Of 1360 progeny of mutagenized F344/NTac males, one male was found to harbor a Lysine-to-Stop change at codon 1137 of the rat *Apc* gene. This mutation causes a truncation of the APC protein at the third amino acid (aa) of the second 15-aa β-catenin-binding domain (Fig. 1) and has a recessive lethal phenotype. As a result, the Pirc line is maintained in heterozygous form as a coisogenic line on F344/NTac, and is commercially available from Taconic.

The KAD rat was developed in collaboration with the Kyoto University Rat Mutant Archive (KURMA), established by ENU-induced mutagenesis of the inbred F344/NSlc rat strain, using the highly effective method of MuT-POWER mutation screening (Mashimo et al., 2008). KAD rats are homozygous for a GC-to-TA transversion mutation in base-pair 7621 of the rat *Apc* gene, leading to truncation after serine 2523 of the APC protein. This truncated APC polypeptide is predicted to retain the β-catenin-binding sites but to lack a part of the basic, EB1-binding and hDLG-binding domains distally to the C-terminus (Fig. 1). This mutation is homozygous viable, presumably owing to the retention of the β-catenin-binding sites. The KAD rat has been backcrossed to the F344/NSlc strain for five generations and the homozygous mutant coisogenic line (F344-*Apc^m1Kyo^*) has been deposited in the National BioResource Project-Rat and is commercially available through Japan SLC, Inc.

Although ~75% of colon cancer cases are sporadic, familial predisposition occurs in a substantial percentage of cases (Burt, 2000). Germline mutations in *Apc* result in the syndrome familial adenomatous polyposis (FAP), in which affected individuals develop hundreds to thousands of polyps as early as in their teens or early twenties (Kinzler and Vogelstein, 1996; Plawski et al., 2013). Individuals with FAP account for only ~1% of all known colon cancer cases, but these individuals have a 100% likelihood of developing cancer unless the colon is removed (Kinzler and Vogelstein, 1996). The location of the mutation within the *Apc* gene can influence the severity of the disease (Fig. 1). Severe polyposis involving hundreds to thousands of polyps is often found in colons of individuals with mutations between codons 1250 and 1464, whereas individuals with mutations proximal to codon 157 or distal to codon 1595 tend to present with far fewer polyps (Nieuwenhuis and Vasen, 2007). Differences in the frequency and type of extra-colonic features, such as desmoid fibromas, also correlate with mutation location. These genotype-phenotype relationships provide insight not only into the disease process itself, but also into the structure and function of the large, multifunctional APC protein complex in other diseases and in normal development.

In this Review, we discuss the biology of each rat strain and describe their experimental features. We go on to provide examples of the ways in which these strains prove useful for understanding the etiology, detection, chemoprevention and treatment of early colon cancer. Finally, we discuss future avenues of colon cancer research that capitalize on the underlying biological features of these rat models in combination with the emergent power of molecular genetics in the rat. The challenge remains to develop more advanced metastatic derivatives of these models.

## Human colon cancer: an introduction

Survival rates of colon cancer patients are highly dependent on the cancer stage at diagnosis. Five-year survival rates are greater than 70% for localized cancer, but plunge below 10% after metastasis to other organs (American Cancer Society, 2014c). About 40% of colonic neoplasms are diagnosed as adenomas before they spread, and another 40% are diagnosed as carcinomas after they have invaded regional lymph nodes; the remaining 20% of cases are diagnosed after the cancer has further metastasized (Howlader et al., 2013). Although early detection can greatly enhance survival, the methods currently in clinical use generally have low compliance and are highly invasive (e.g. colonoscopy). Furthermore, some diagnostic methods are either non-specific or lack sensitivity for the earliest operable stages. Thus, new methods for chemoprevention and early detection that are less invasive and more accessible must be developed to reduce the worldwide burden of this disease. Appropriate animal models for the early stages of colon cancer can uniquely contribute to reaching this goal.

As demonstrated by the wide variability in the incidence of colon cancer around the world, environmental factors are likely to significantly impact the risk of an individual developing this disease. One pertinent example is the association between inflammation and the subsequent development of colon cancer. Individuals with longstanding ulcerative colitis, an inflammatory disease of the large bowel, are three to four times more likely to develop colorectal cancer during their lifetime, compared with those without an inflammatory bowel disease (Eaden et al., 2001). These individuals also tend to have poorly differentiated carcinomas, which lead to a worse prognosis (Mikami et al., 2011). This link between inflammation and colon cancer is further supported by the association between the use of the anti-inflammatory compound aspirin and reduced colon cancer risk (Chan et al., 2012). Thus, bowel inflammation presents a potentially important target for intervention in colon cancer.

For individuals with a history of adenomatous polyps or cancer, chemoprevention is especially important to prevent recurrence (American Cancer Society, 2014b). Dietary factors, such as fiber intake and vitamin D supplementation, are currently being studied in both humans and in animal models to determine their effectiveness in preventing the recurrence of colonic adenomas. However, these studies are difficult to carry out in humans because variable dietary and lifestyle factors might affect results. Studies of chemoprevention must consider many different issues: primary prevention versus recurrence, prevention versus treatment, the molecular pathway along which the tumor developed, and the dose and duration of treatment needed to be effective. This last point is especially important in considering tests in animal models, because colon cancer has a long latent period (Jones et al., 2008) and a successful preventive agent might need to be in place far in advance of carcinoma establishment.

Although effective chemopreventive measures can reduce the incidence of colon cancer, the burden in human populations can be reduced most effectively by detection at an early, operable stage. Several methods are under development to complement the gold standard of colonoscopy. Computed tomographic colonography (CTC) is non-invasive but nonetheless requires clearance of the bowel and, when a tumor is detected by CTC, the patient must then undergo traditional colonoscopy and polypectomy. A new method to detect methylated DNA in stool does not require the bowel clearance step that limits the compliance of colonoscopy and CTC. This method currently can detect 95% of advanced cancers with 90% biological specificity (Lidgard et al., 2013), but is unreliable for detecting early adenomas. Markers based on blood or urine samples would increase compliance compared with stool tests; thus, ELISA methods are being sought to detect altered proteins in serum and plasma (Ladd et al., 2012; Lin et al., 2013). However, ELISA methods rely on the availability of an antibody specific to their target of interest. By contrast, urinary metabolomics using nuclear magnetic resonance (NMR) spectroscopy does not require antibodies, although here too the sensitivity and specificity for detecting early adenomas is less than 65% (Eisner et al., 2013). Mass spectrometric analysis of metabolomic pathways that are altered in the urine of animal models and humans with colonic adenomas might increase the sensitivity and specificity for detecting these earliest operable lesions (Manna et al., 2014).

Without a comprehensive understanding of all of the pathways and fates of early and late stages of cancer, cancer heterogeneity can limit early detection. It is now recognized that adenomas vary in fate, with some growing, remaining static or even spontaneously regressing (Pickhardt et al., 2013), and that cancers occurring under different genetic or environmental conditions might require different modalities of prevention, detection or treatment. The Cancer Genome Project is identifying numerous putative driver and passenger mutations in each cancer type (http://cancergenome.nih.gov/), and the discovery of treatments based on these findings will require the identification of therapeutic targets *in silico*, followed by testing *in vitro* and subsequently in appropriate *in vivo* models. Thus, without physiologically relevant *in vivo* models in which to investigate newly identified cancer molecular pathways, efforts to develop new therapies leading to early clinical trials will continue to have limited success.

## Rodent models of colon cancer

There are two main factors that influence an organism’s suitability as a human disease model: its similarity to the physiology and disease in the human, and the ease with which it can be genetically and experimentally manipulated. Recently, the genetic resources available for the rat have been developed to keep pace with those of the mouse. Full sequencing of the genomes of several laboratory rat strains has made this possible (Guo et al., 2013; Worthey et al., 2010). Additionally, new genome-editing technologies such as ZFNs (Cui et al., 2011; Geurts et al., 2009; Mashimo et al., 2010), TALENs (Mashimo et al., 2013; Ponce de León et al., 2014) and CRISPR/Cas (Li et al., 2013a; Li et al., 2013b; Ma et al., 2014; Mashimo, 2014; Yoshimi et al., 2014) can now be used to alter the function of specific rat genes. Until the very recent advent of these methods of directed mutagenesis, rat mutational strategies were limited to spontaneous mutants (Eker and Mossige, 1961) or random germline mutagenesis by N-ethyl-N-nitrosourea (ENU) (Mashimo et al., 2008) accompanied by target selection (Smits et al., 2006; Zan et al., 2003).

By ENU germline mutagenesis, the *Apc^Min/+^* mouse (which develops multiple intestinal adenomas owing to a point mutation in the *Apc* gene) was discovered over two decades ago (Moser et al., 1990). Although this mouse model offers many genetic advantages for studying intestinal cancer (Gondo, 2013; Oshima and Oshima, 2012; Washington et al., 2013; Young et al., 2013; Zeineldin and Neufeld, 2013), it fails to model human colorectal cancer in that the majority of tumors in this mouse arise in the small intestine rather than in the colon (on the standard C57BL/6J background, *Apc^Min/+^* mice develop ~100 adenomas in the small intestine and only 0–2 adenomas in the colon) (Moser et al., 1990). In the human, tumors of the small intestine occur far less often than in the colon (Preston et al., 2008).

**Fig. 2.**
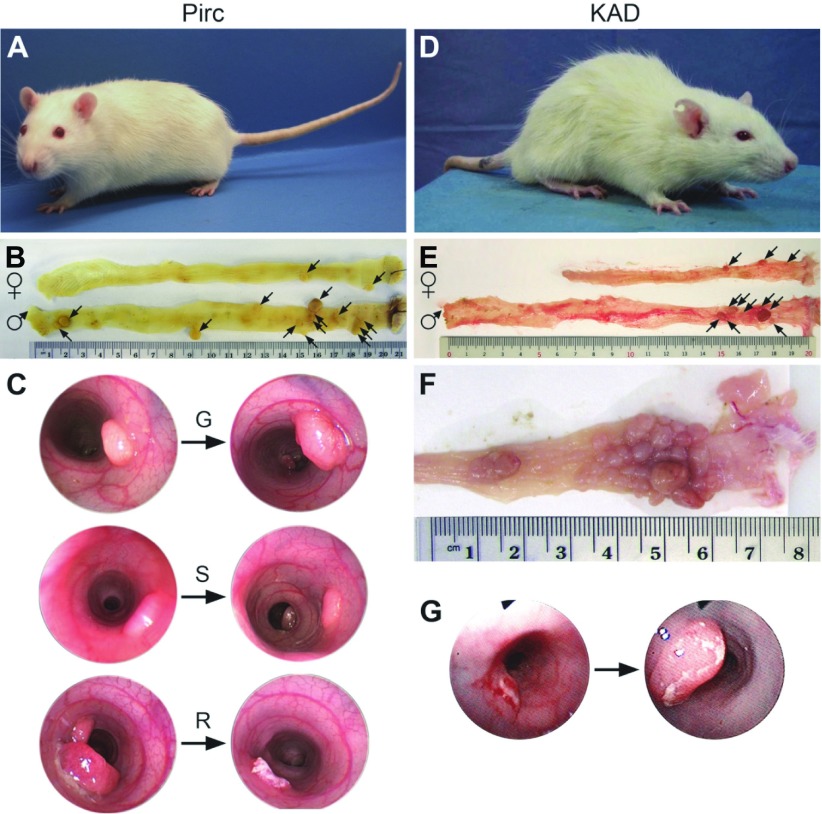
**Features of the *Apc* mutant rat models.** (A) The Pirc rat, which develops intestinal tumors spontaneously, with tumor development biased towards the colon. (B) Colons from adult female (top) and male (bottom) Pirc rats (210 days of age), demonstrating a gender bias in colon tumor multiplicity, with females developing fewer tumors than males. Tumors are indicated by arrows. (C) Colonoscopy images from adult Pirc rats, demonstrating the distribution of colonic tumor fates: G, growing; S, static; R, regressing. (D) The KAD rat, which does not develop spontaneous tumors but tumors do develop in response to treatment with AOM and DSS. (E) Colons of adult KAD rats treated with AOM/DSS, which also show a gender bias in colon tumor multiplicity (15-weeks post-treatment), with females (top) developing fewer tumors than males. Tumors are indicated by arrows. (F) Colon of an adult AOM/DSS-treated KAD rat, showing the characteristic development of large clusters of tumors in the distal colon (20-weeks post-treatment). (G) Endoscopy shows that most tumors in the KAD rat are growing, as illustrated by this example. AOM, azoxymethane; DSS, dextran sodium sulfate.

By contrast, the heterozygous *Apc^Pirc/+^* (Pirc) rat develops colonic tumors spontaneously at a high frequency (Amos-Landgraf et al., 2007). A second *Apc*-mutant rat (KAD) is homozygous, viable and does not develop tumors spontaneously but has enhanced susceptibility to agents that promote the development of colonic tumors (Yoshimi et al., 2009). Thus, these two complementary rat models allow the study of both sporadic and environmentally induced cancers arising preferentially in the colon (Fig. 2). The difference between these models also illustrates how having more than one mutant allele of *Apc* can help to dissect the functions of this multifaceted gene.

Tumor multiplicity data forms the basis for the majority of investigations into modifiers of intestinal cancer risk. Although this measure provides important quantitative information, it limits understanding to a static point in time. Beyond this, longitudinal studies utilizing colonic endoscopy provide more specific qualitative and quantitative information as relates to time. For this reason, the statistical power to study the colonic region in these rat models exceeds that of the *Apc^Min/+^* mouse. Although colonic tumorigenesis can be increased in mouse models, it requires further genetic manipulation, carcinogenic treatments, or chemical or physical abrasion (Byun et al., 2014; Grill et al., 2014; Hinoi et al., 2007; Hung et al., 2010; Møllersen et al., 2004; Paul Olson et al., 2014; Ritchie et al., 2009; Suzui et al., 2002).

For more information on how these two rat models were created, please refer to Box 2. Below we discuss in more detail the current understanding of the development of colon cancer in the Pirc and KAD rat models. Finally, we discuss the current limitations and avenues for further

## Cancer development in the Pirc and KAD strains

**Fig. 3.**
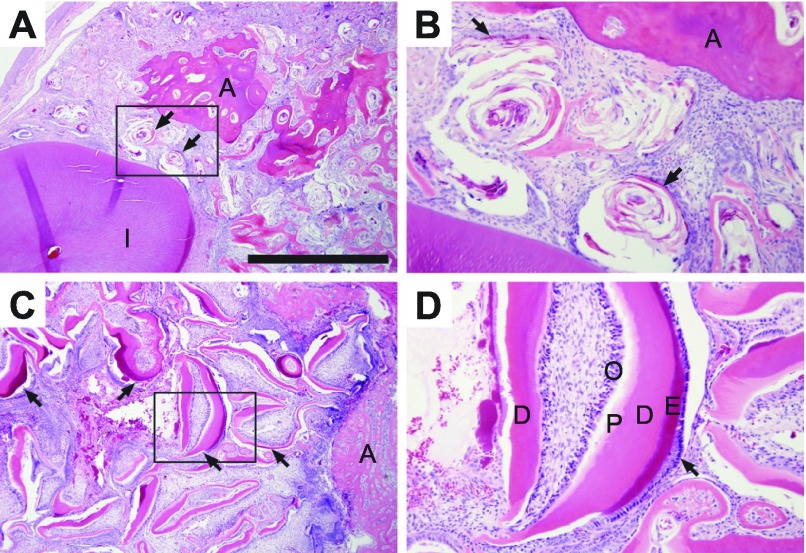
**Complex odontoma associated with F344/NTac-Pirc rats.** (A) Complex odontoma with moderately disorganized nests of epithelial and mesenchymal components, with flattened epithelium forming cyst-like structures with layered eosinophilic material (arrows) located at base of lower incisors (l) with alveolar bone (‘A’) at the periphery of the mass. (B) Magnification (4×) of boxed area in A, showing nests of epithelial and mesenchymal cells (arrows). (C) Complex odontoma with organized, primitive tooth structures (arrows) with distinct layers of well-defined tooth structures and alveolar bone at mass periphery. (D) Magnification (4×) of boxed area in C, showing primitive tooth structures with distinct well-defined tooth layers, including odontoblasts (O), predentin (P), dentin (D), enamel (E) and ameloblasts (arrow). Scale bar: 1 mm (A,C).

Pirc rats have a unique biology that mirrors many features of human colon cancer. Reflecting recent studies of human populations (Brenner et al., 2007; Murphy et al., 2011; Regula et al., 2006), tumors in Pirc rats occur earlier in males than in females (as early as 45 days of age in males, whereas it can take weeks longer to appear in females) (Fig. 2). This gender disparity extends to tumor multiplicity and time to morbidity. At 7 months (210 days) of age, males develop 20.4±8.9 tumors in the colon and 17.0±7.3 tumors in the small intestine, whereas females develop only 9.2±6.0 colonic tumors and 1.9±1.6 small intestinal tumors, both with 100% penetrance (Table 1 and references therein). Males become moribund at approximately 11 months of age owing to rectal bleeding, anemia, weight loss and intestinal blockage. In surviving older animals, secondary conditions such as jaw odontomas (dental tumors) are common (Fig. 3), although males generally become moribund before developing these additional tumors. This odontoma phenotype, a hallmark of FAP in humans (Gardner, 1962; Gardner and Plenk, 1952), is incompletely penetrant in the Pirc rat, but has not been reported in mouse models.

The inflammatory aspect of colon cancer can be modeled in rodents using the ulcerogenic and inflammatory agent dextran sodium sulfate (DSS) to induce experimental colitis (Hirono et al., 1981). Although both Pirc and KAD rat models show enhanced susceptibility to agents that induce inflammation, the two models contribute in different ways to our understanding of inflammatory processes and their effect on colon cancer risk and progression. F_1_ derivatives of the Pirc rat involving the sensitive strain ACI have been given 4% DSS (500 kDa) in the drinking water for two 7-day treatments, separated by 1 week without DSS. Colonic tumorigenesis is enhanced over the spontaneous level by this exposure to DSS (Table 1) (Irving et al., 2011): at 140 days of age, DSS treatment increases colonic multiplicity sixfold in females (45.7±10.2 versus 7.5±3.4 in controls, Wilcoxon rank sum test *P*<0.007) and more than twofold in males (56.3±3.5 versus 22.1±6.1 in controls, *P*<0.0001). However, DSS treatment does not significantly affect tumor multiplicity in the small intestine (*P*=0.51 for females and *P*=0.35 for males).

**Fig. 4.**
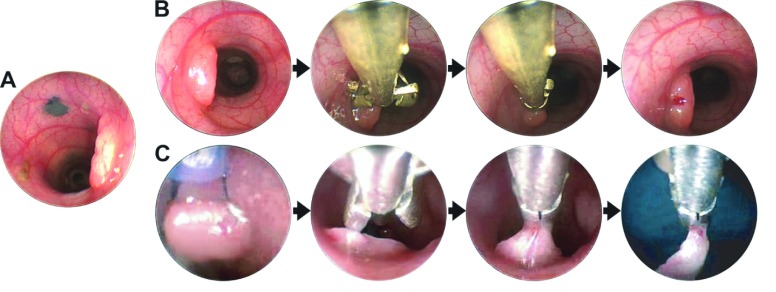
**Endoscopic procedures in rats.** (A) *In vivo* tumor tattooing. Colonic tumors and normal tissue can be marked using a surgical sclerotherapy needle and sterile India ink. Tattoos are made in the submucosal layer for the long-term tracking of tumors for longitudinal analyses or to monitor recurrence at polypectomy sites. (B) *In vivo* biopsy. Rats are anesthetized and normal or tumor tissue is biopsied using biopsy forceps. (C) *In vivo* polypectomy and recovery of tumors. Whole tumors can be removed by electrosurgical polypectomy using a standard pediatric-sized snare cautery.

Beyond tumor multiplicity, other facets of tumor development in these rat models can deepen our understanding of the early adenoma. In patients followed longitudinally by CTC, the majority of macroscopic colonic tumors became static and did not grow (Pickhardt et al., 2013). Importantly, the tumors that did grow were highly correlated with neoplasms that were later classified histologically as advanced-stage adenomas. The Pirc rat model shows a distribution of fates similar to the human: in these rats, on average, 71% of adenomas grow, whereas 25% are static and 4% regress (Irving et al., 2011; Irving, 2014). This important aspect of tumor biology warrants further investigation. India ink can be used during endoscopy to facilitate identification and precise tracking of specific tumors *in vivo* (Fig. 4A).

The KAD rat model provides a useful contrast to the Pirc rat and also to all known mouse models of intestinal neoplasia. Uniquely, both sexes of the KAD rat can survive more than 1.5 years without any spontaneous development of tumors, despite homozygosity for the KAD mutation. KAD rats express the truncated form of APC polypeptide at a level similar to that of normal APC levels in wild-type F344 rats (Yoshimi et al., 2009). As a result, unlike other rodent models mutated in *Apc*, WNT signaling in the KAD rat remains intact. In support of this, WNT3A ligand is able to increase WNT activity in fibroblasts derived from KAD rats to a similar degree as in those derived from wild-type rats (Yoshimi et al., 2013). However, the formation of colonic tumors in KAD rats depends completely on inflammation (see Box 3). Whereas DSS treatment alone causes colonic inflammation, when KAD rats are also given a single subcutaneous dose of the carcinogen azoxymethane (AOM; 20 mg/kg body weight) followed 1 week later by 2% DSS (36–50 kDa) in the drinking water for 7 days (AOM/DSS treatment), colonic tumors form in 100% of these animals (Yoshimi et al., 2009) (Table 1).

**Box 3. KAD rats and colonic inflammation**

In the KAD rat, the distal part of the APC C-terminus is lost. KAD rats sustain chronic colonic inflammation post-DSS-treatment much longer than wild-type rats (Yoshimi et al., 2013). The inflamed KAD colon shows mucosal ulceration, crypt loss, edema, diffuse inflammatory cell infiltration of the lamina propria and submucosa, congestion and dilation of local capillary blood vessels, elevated expression of *Cox2* and *Ptges*, and enhanced cell proliferation within the intestinal mucosa. The damaged mucosal epithelium is deficient in fibrin clots and shows reduced angiogenesis typically associated with wound healing. Expression of *Apc* is upregulated in the vascular endothelial cells (VECs) of the inflamed colonic region, suggesting an association between the C-terminus of APC and angiogenic mucosal repair (Yoshimi et al., 2013). DLG5 function is lost in the KAD rat; this protein is also defective in inflammatory bowel disease (IBD) (McGovern et al., 2010; Yamazaki et al., 2004), suggesting an association between APC and IBD. Thus, the KAD rat pinpoints a function of APC in the regulation of inflamed colonic states, separate from the tumor-suppressing functions encoded proximally to the C-terminus.

As is seen in humans and the Pirc rat, AOM/DSS-treated KAD rats also exhibit a gender bias in colonic tumorigenesis: compared with females, male rats develop significantly more (9.5±1.8 versus 5.8±2.3 in females, Student’s *t*-test *P*<0.01) and larger (33.9±23.0 mm^3^ versus 10.1±8.3 mm^3^ in females, *P*<0.02) colonic tumors (Yoshimi et al., 2012) (Table 1). At 15 weeks post-treatment, AOM/DSS-treated KAD males have sevenfold more colonic tumors than AOM/DSS-treated wild-type rats (9.5±1.8 versus 1.3±0.8, *P*<0.0001), whereas the average number of colonic tumors in AOM/DSS-treated KAD females only doubles (5.8±2.3 versus 3.8±3.3, *P*=0.25). These AOM/DSS-treated male KAD rats become moribund between 28 and 33 weeks after treatment, owing to rectal bleeding, anemia and weight loss. The distribution of tumors along the colon of treated KAD males is similar to that seen in human patients, occurring more frequently in the rectum and distal colon than in the middle colon. Although tumors from KAD rats show nuclear localization of β-catenin protein, the β-catenin-binding domains of APC remain intact, even in developed tumors. Instead, the majority (about 75%) of tumors from AOM/DSS-treated KAD rats contain missense mutations in the β-catenin gene itself. These observations indicate that the signaling pathway downstream of WNT is constitutively activated by β-catenin in the tumors of the KAD rat treated with DSS and AOM (Yoshimi et al., 2009). As discussed above, this pathway includes tumor-promoting functions such as those of MYC. In addition to adenoma development, about one third of the colonic tumors in AOM/DSS-treated KAD rats are differentiated tubular adenocarcinomas. These tumors invade layers of intestinal tissue that underlie the epithelium, including the submucosa, muscularis propria and serosa. High-resolution images of example tumors from both Pirc and KAD rats at various stages can be viewed at the Murine Models of Gastrointestinal Tumors virtual slide box at http://mmgint.org/.

Because no metastasis to other sites has been observed in the colonic tumors of the AOM/DSS-treated KAD rat (Yoshimi et al., 2009), these advanced but localized colonic neoplasms represent operable stages that can be used to assess strategies for the early detection of surgically curable colonic cancers.

**Table 1.**
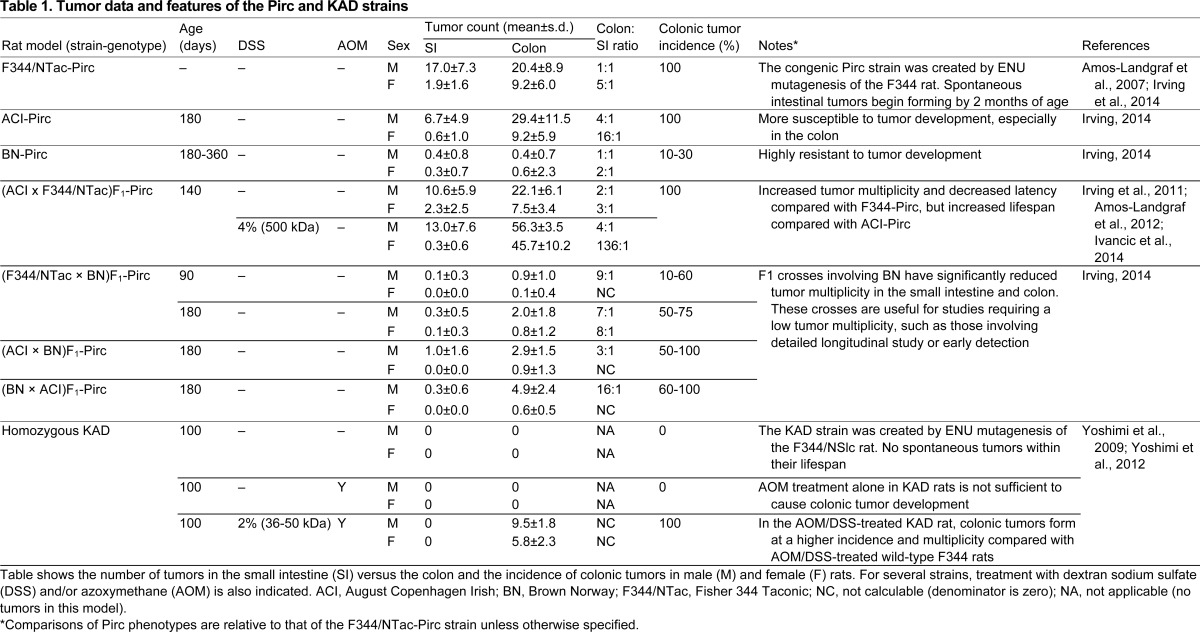
Tumor data and features of the Pirc and KAD strains

## Genetics of the Pirc strain: genotype and phenotype

Along with the differences in intestinal cancer phenotype, the mouse and rat differ in their genomic structure, which impacts the study of colon cancer. Although these two genera are about 93% identical in nucleotide and amino acid sequence (Wolfe and Sharp, 1993), the organization of the rat genome is similar to that of the human in an important way (Zhao et al., 2004): mouse chromosomes are telocentric, with their centromeres lying near the end of each chromosome, whereas the centromere of most chromosomes in the rat and the human lies near the middle of its chromosome (metacentric chromosomes) (Larsson et al., 1998). Thus, the availability of markers on both sides of the centromere permits one to discriminate between different classes of chromosomal rearrangements in the disease process: loss, translocation or homologous mitotic recombination (Amos-Landgraf et al., 2007; Haigis and Dove, 2002).

The coisogenic F344/NTac-Pirc strain has been crossed to several inbred rat strains and outbred stocks to determine the effect of genetic background on its tumor phenotype (Table 1). F_1_-generation animals between F344/NTac-Pirc rats and most other inbred genetic backgrounds, including Brown Norway (BN) and DahlSS, have fewer tumors in both the small intestine and colon compared with the F344/NTac-Pirc parent. By contrast, F_1_ animals between the congenic ACI-Pirc and F344/NTac have more tumors relative to the F344/NTac-Pirc parent, as well as decreased latency and an enhanced bias toward the colon versus the small intestine.

The variation in tumor phenotype provided by these F_1_ hybrids has proven useful for certain investigations. On one hand, the high tumor numbers in (ACI × F344/NTac)F_1_-Pirc hybrids has enhanced the investigation of vitamin D compounds in both chemoprevention of newly emerging tumors and therapy of existing tumors (Irving et al., 2011). On the other hand, the dramatic decrease in tumor numbers and penetrance of (F344/NTac × BN)F_1_-Pirc hybrids enables extended longitudinal studies of single, isolated adenomas. In these hybrids, at 3 months of age only 10% of females and 60% of males develop even a single tumor, whereas, by 6 months of age, the penetrance rises to 50% in females and 75% in males (Table 1).

It is important to recognize that F_1_ animals provide a uniform background with informative polymorphisms between the parental strains across the genome. Thus, one can identify loss of heterozygosity at DNA loci throughout the tumor genome, thereby revealing genetic changes that drive tumor development. Furthermore, by genotyping polymorphic markers in both genomic DNA and cDNA from heterozygous F_1_ Pirc rats, some tumors have been shown to involve loss of the wild-type allele at the *Apc* locus, whereas others involved only loss of expression of the wild-type *Apc* allele (Amos-Landgraf et al., 2012). This latter case could reflect stochastic epigenetic silencing at the *Apc* locus (Amos-Landgraf et al., 2012). Such monoallelic differences in expression are becoming increasingly apparent in the genetic etiology of cancer and could be valuable in deciphering the role of regulatory variants that lie within promoters and enhancers (Valle et al., 2008; Yan et al., 2002). Monoallelic expression in tumors is a hallmark of a tumor suppressor locus with a haploinsufficient phenotype (Berger et al., 2011; Largaespada, 2001; Payne and Kemp, 2005).

The genetic description of sporadic colon cancer development has largely been derived from human tumors harvested at various stages. Although such analyses have provided statistical criteria by which to define driver mutations that can promote tumor progression, they limit the perspective to single points in time. The Pirc rat provides an opportunity to longitudinally follow the emergence, growth and progression of individually annotated tumors using endoscopy and a newly developed method of measuring tumor volume by gel-molding (Irving et al., 2014). Thus, molecular changes in tumors can be examined in endoscopic biopsies (Fig. 4B), enabling quantitative observations of tumor development to be combined with molecular analysis. This, in turn, enriches the study of response to treatment.

## Investigating the biology of colon cancer by using the Pirc and KAD rat models

Here, we discuss in more detail how these rat models can be used for the longitudinal study of the etiology, early detection, chemoprevention and treatment of colon cancer.

### Tumor etiology

As evidenced by the varying global distribution of risk of and mortality from colon cancer, tumor etiology likely influences cancer progression and thus viable treatment options. By comparing tumors in sporadic models to those induced by inflammation, we can begin to understand how etiology affects their longitudinal fate. The Pirc and DSS-Pirc models allow investigation of this issue.

Colonic tumors from Pirc rats are histologically classified into three broad categories: adenoma (neoplastic cells confined to the mucosal layer), intramucosal carcinoma (invasion of neoplastic cells into the lamina propria or into but not through the muscularis mucosa) and early carcinoma (invasion of neoplastic cells through the muscularis mucosa and into but not through the submucosa) (Washington et al., 2013). Although tumor multiplicity is increased in both female and male Pirc rats following DSS treatment, males differ from females by also showing more advanced stages (Irving, 2014). In 180-day-old untreated females, there are no early carcinomas, 38% are intramucosal carcinomas and 63% are adenomas; these proportions are not significantly altered by DSS treatment (0%, 46% and 54%, respectively, chi-squared test *P*=0.48). Similarly, at 160 days of age, untreated males have no early carcinomas, only 18% are intramucosal carcinomas and 82% are adenomas. In contrast to females, DSS treatment of males significantly enhances tumor stage: 41% are early carcinomas, 36% are intramucosal carcinomas and only 23% are adenomas (*P*<0.00001). Furthermore, although untreated aged males can develop adenocarcinomas, DSS treatment leads to adenocarcinomas at an earlier age (Washington et al., 2013). Notably, unlike the mouse models of colon cancer, the involvement of stroma in Pirc rat colon tumors is similar to that seen in humans (Mary Kay Washington, Department of Pathology, Microbiology and Immunology at Vanderbilt University Medical Center, personal communication to W.F.D.).

### Early detection

The rat is a better biological model for developing early detection strategies owing to many limitations in the mouse. For example, the size of the rat permits larger samples, enabling multiple assays from a single sample. New methods for detecting tumors in easily obtained biological tissues and fluids are currently being investigated. Although the use of such markers on human samples is the ultimate goal, identifying potential known markers is often hindered by the difficulty in overcoming genetic and environmental variability between individuals. Studies in the inbred *Apc^Min/+^* mouse have addressed this by using reciprocal metabolic labeling to provide mixed proteome samples from mutant versus wild-type littermates (Huttlin et al., 2009; Ivancic et al., 2013). However, in this strain the predominance of tumors in the small intestine confounds the search for markers specific to colonic tumors. To address this problem, the Pirc rat has been utilized to validate serum markers of colonic tumors that are differentially expressed in rat tumor tissue or in tissue or serum from the *Apc^Min/+^* mouse. From the proteomic analysis of serum proteins from mutant versus wild-type rats, EGFR, LRG1, ITIH4 and F5 were identified as biomarkers that together can detect early adenomas in the Pirc rat (Ivancic et al., 2014). However, these biomarkers might originate from tissues other than the tumor. Therefore, it is necessary to control for the possibility that the constitutional *Pirc/+* genotype of the broadly expressed *Apc* gene produces pleiotropic phenotypic effects, off-target with respect to colonic neoplasia. The resistant (F344/NTac × BN)F_1_-Pirc strain might thus prove useful because it carries few colonic tumors and some *Pirc/+* progeny remain tumor-free. It will be possible to identify markers specific to the presence of a tumor by comparing tissues or body fluids from cohorts of tumor-positive (F344/NTac × BN)F_1_-Pirc rats versus samples from cohorts of genetically and environmentally matched but tumor-negative rats. Further enhancing these studies, the gel-molding method (Irving et al., 2014) can document the growth profile of each colonic tumor, permitting the specific assignment of any signals corresponding to the growing adenoma.

### Chemoprevention and treatment

Owing to their colonic phenotype as well as their high tumor multiplicity and large body size, the Pirc and KAD models have been useful for chemoprevention and treatment studies. When Pirc rats were treated with the nonsteroidal anti-inflammatory agent celecoxib for 5–6 months, colonic tumor multiplicity was significantly reduced in both males and females (Amos-Landgraf et al., 2007). However, no significant effect was observed following aspirin treatment for 3–4 months (Irving, 2014). This difference could be caused by the length of treatment or to different actions of these agents: celecoxib is a selective inhibitor of cyclooxygenase-2 (Dutta et al., 2009), whereas aspirin is a non-selective inhibitor of both cyclooxygenase-1 and cyclooxygenase-2 (Vane and Botting, 2003).

A challenge for any chemopreventive study is to accurately monitor *in vivo* changes in tumor multiplicity, size and gene expression over time. A longitudinal study design enables tests of compounds against newly arising tumors (prevention), as well as against established tumors (treatment). Compared with traditional cross-sectional studies in which subsets of animals are sacrificed at different time points, the statistical power of longitudinal studies is increased by following multiple annotated tumors per animal. Although small-scale computed tomography (microCT) can be used to assess tumors in living mice and rats (Weichert, 2004), the equipment required is costly and investigators must often share these facilities with animals coming from different levels of biosafety and pathogen status. These logistical limitations compromise the use of these specialized imaging facilities for longitudinal studies. An alternative method has been developed for objectively assessing colonic tumor development in living rats that is not hampered by these limitations. This method involves the molding of tumors using biocompatible alginate gel (Irving et al., 2014). Experimental groups are established based on the number of tumors observed by endoscopy and then followed by tracking tumor growth over time. Using a similar but more subjective approach, it has been shown that supplementing Pirc rats with several different vitamin D compounds neither reduced tumor multiplicity nor altered tumor growth patterns in the colon (Irving et al., 2011). Similarly, recent evidence in humans suggests that levels of vitamin D above that sufficient for bone health is not associated with reduced cancer risk (Bikle, 2014). Before studies of possible candidates for chemoprevention by dietary supplementation are expanded in human populations, the experimental advantages discussed above make the rat the preferred platform for definitive early studies.

Endoscopic methods have also been used to group together subsets of KAD rats with known numbers of colonic tumors prior to treating them with chemotherapeutics (Yoshimi et al., 2009; Yoshimi et al., 2012; Kochi et al., 2014). Treatment of tumor-bearing KAD rats with the established chemotherapeutic drug 5-fluorouracil (5-FU) significantly reduces the size of adenocarcinomas, and results in a reduction in cell proliferation and an increase in apoptosis (Yoshimi et al., 2012). As with human patients, diarrhea and weight loss were observed as side effects of 5-FU treatment. Thus, overall, many tumor features and treatment responses in the KAD rat are similar to those reported in the clinic for human colon cancer. Consequently, these models can effectively promote the development of preventive and treatment strategies for human colorectal cancer.

## Conclusions and future developments

In this Review, we have identified ways in which the understanding of the etiology, early detection, chemoprevention and treatment of colon cancer can be developed further using rat models. The importance of further developing the experimental resources in the rat for modeling human colon cancer began with the initial finding that *Apc* deficiency in the rat leads primarily to colonic tumors. This result, unlike the distribution found in the *Apc^Min/+^* mouse, accurately reflects that in the human patient. Furthermore, modeling of colon cancer in the rat introduces the opportunity to analyze the genetic and epigenetic etiology of tumors, afforded by its metacentric karyotype. Finally, the size of the rat compared to the mouse facilitates tumor detection and monitoring of tumor fate by endoscopy and gel-molding. Until recently, the genetic resources created for the laboratory mouse eclipsed the physiological reasons for using the rat. However, it is now feasible to carry out targeted genomic editing in the rat (Cui et al., 2011; Geurts et al., 2009; Li et al., 2013a; Li et al., 2013b; Ma et al., 2014; Mashimo et al., 2008; Mashimo et al., 2010; Mashimo, 2014; Yoshimi et al., 2014).

The next generation of genetic analysis of colon cancer biology in experimental mammalian models involves further dissection of the multiple functions of the *Apc* gene, as well as a genome-wide discovery of interacting factors through modifier genetics. Technically, these analyses can follow parallel paths in the mouse and the rat. One can expect further improvements in the colonic phenotype of *Apc*-related mouse models. An understanding of human colon cancer will depend on the extent to which a manipulable rodent model simulates the corresponding biology of the human (Seok et al., 2013).

The multiple functions of APC can be dissected by targeted allelic substitutions using the new methods of genomic editing cited above. It is an important priority for both the mouse and the rat to enrich the set of mutant *Apc* alleles beyond truncating nonsense alleles that currently dominate the spectrum in the human and in rodent models. Truncations remove all functions encoded by regions distal to the mutation. Molecular interaction studies have identified numerous candidate regions for functionality in the C-terminal region of the protein (Nelson and Näthke, 2013). The results from the investigations of the two distinct alleles of *Apc* in the Pirc and KAD rat models support the use of targeted replacement and alternative knockouts in specific domains to investigate functions of *Apc* (Yoshimi et al., 2014). For example, the ability to construct specific knock-in mutant alleles of the *Apc* gene in the mouse has enabled Neufeld and colleagues to assess the importance for WNT signaling of the apparently redundant nuclear localization signals in codons 1769–1772 and 2048–2053 (Zeineldin et al., 2012).

The strong effects of genetic background on the Pirc phenotype can be dissected by the methods of quantitative trait genetics. Thanks to the efficient ENU mutagenesis of the rat germline cited above, the genetic system impacting the biology of the Pirc strain can be extended by mutagenesis beyond the viable polymorphic alleles present in inbred strains (Dove et al., 2014). Compared with the *Modifier-of-Min* loci of the mouse, Pirc modifiers affecting colonic tumors are more likely to be orthologous to such loci discovered by genome-wide association analyses in human populations (Dunlop et al., 2012; Peters et al., 2013). The genetic control of the Pirc phenotype resulting from strain variation is eclipsed only by the strong differences between sexes within each genetic background. This gender effect is sufficiently strong in the rat to enable an investigation of its mechanism by hormonal and genetic manipulation.

The stage of metastasis is a crucial biological event that remains largely elusive in the modeling of colon cancer. Some progress has been reported in the mouse toward establishing apparent metastatic lesions in the liver (Hung et al., 2010; Jackson et al., 2001) and in the lymph nodes and lung (Trobridge et al., 2009). However, a rigorous documentation of metastasis, in the mouse or the rat, will depend on the ability to perform lineage tracing of sporadic tumors (see Washington et al., 2013).

Beyond these avenues along which the rat can broaden our understanding of colon cancer, a major vista for future advances involves the intersection of cancer with mammalian neurobiological processes. This is exemplified by the classic study of chronotherapy, in which the time of day was found to influence tolerance to chemotherapeutic treatments (Halberg et al., 1973). Patterned chronotherapy is now being investigated in mouse models to determine optimal timing to enhance effectiveness while decreasing the side effects of the treatment (Li et al., 2013c). Mutation of the key circadian gene *Per2* has been found to influence tumor development in the *Apc^Min/+^* mouse model (Wood et al., 2008), supporting a role for neurobiology in oncogenesis. The laboratory rat has a rich history of neurobiological investigations to draw upon. For example, it has been shown that the rat has a much higher level of brain function compared with the mouse, with recognized metacognition and altruism (Foote and Crystal, 2007; Rutte and Taborsky, 2007; Zhang et al., 2013). Additionally, the gut-brain axis is increasingly being recognized as a modulator of stress responses (Bhatia and Tandon, 2005). Rats also exhibit complex behavioral patterns that are influenced by maternal care to establish behaviors that persist throughout the life of the rat. These behaviors in turn have been shown to influence hormone production and alter stress responses throughout life (Zhang et al., 2013). This exemplifies the persistent changes that are established through differences in behavior and can potentially influence disease progression (Kosten et al., 2014). Recent publications indicating a direct link between the microbiota and their generated metabolites demonstrate direct influences on behavior and immune responses (Barouei et al., 2012; Crumeyrolle-Arias et al., 2014; De Vadder et al., 2014). Because of its rich biology, the rat is now poised to reveal some of the complex interactions among the gut, brain and the microbiota that influence behavior, stress, immune responses and cancer.
